# Identification of Potential Core Genes in Parkinson's Disease Using Bioinformatics Analysis

**DOI:** 10.1155/2021/1690341

**Published:** 2021-09-17

**Authors:** Wei Quan, Jia Li, Xiya Jin, Li Liu, Qinghui Zhang, Yidan Qin, Xiaochen Pei, Jiajun Chen

**Affiliations:** Department of Neurology, China-Japan Union Hospital of Jilin University, No. 126, Xian Tai Road, Changchun, Jilin 130000, China

## Abstract

**Purpose:**

This study aimed to explore new core genes related to the occurrence of Parkinson's disease (PD) and core genes that can lead to the progression of PD.

**Methods:**

The expression profile data of GSE42966, which contained six substantia nigra tissues isolated from normal individuals and nine substantia nigra tissues isolated from patients with PD, were obtained from Gene Expression Omnibus. Differentially expressed genes (DEGs) were identified, followed by functional enrichment analysis and protein-protein interaction (PPI) network construction. We then identified 10 hub genes and analyzed their expression in different Braak stages.

**Results:**

A total of 773 DEGs were identified that were significantly enriched in metabolic pathways. Ten hub genes were identified through the PPI network, namely, *GNG3*, *MAPK1*, *FPR1*, *ATP5B*, *GNG2*, *PRKACA*, *HRAS*, *HSPA8*, *PSAP*, and *GABBR2*. The expression of *HRAS* was different in patients with PD with Braak stages 3 and 4.

**Conclusion:**

These 10 hub genes and the metabolic pathways they are enriched in may be involved in the pathogenesis of PD. *HRAS* may have potential value in predicting the progression of PD.

## 1. Introduction

Parkinson's disease (PD) is a severe neurological disease resulting from the progressive degeneration of dopaminergic neurons located in the substantia nigra [1]. As the second most common neurodegenerative disease, PD affects approximately seven million people worldwide [2]. Currently, this disease has no cure. As the disease progresses, the patient's motor and nonmotor symptoms gradually aggravate, which seriously affects the quality of life in elderly patients.

Current research suggests that PD is caused by a combination of genetic and nongenetic factors [3]. Although researchers have confirmed that many genes are related to the occurrence of PD, including *LRRK2* [4] and *VPS35* [5], the specific pathogenesis is still unclear. There is a lack of understanding of the key genes that can identify the progression of PD. Therefore, it is important to identify new genes potentially involved in the manifestation and progression of PD. These genes could serve as possible new targets for the treatment of PD.

Our research aims to discover new key genes related to the occurrence of PD and core genes involved in the progression of PD. To identify differentially expressed genes (DEGs) between the substantia nigra of patients with PD and healthy individuals, bioinformatics methods were used to analyze gene expression profiling data downloaded from the Gene Expression Omnibus (GEO) database. Gene Ontology (GO) functional annotation analysis and Kyoto Encyclopedia of Genes and Genomes (KEGG) pathway enrichment analyses were performed for the DEGs identified. We then established a protein-protein interaction (PPI) network to identify hub genes related to PD. Finally, we analyzed the expression of the hub genes in patients with PD with Braak stages 3 and 4. Braak staging is based on a hypothesis on the cause of sporadic PD proposed by Braak in 2003, which states that an unknown pathogen in the intestine may be the cause of PD. Based on the specific mode of *α*-synuclein delivery, they proposed a PD-related pathological staging system, termed as Braak staging. Braak staging is divided into six stages according to the degree of brain involvement in patients with PD. The gradual expansion of the disease from stage 1 to stage 6 also indicates the progression of the disease [6, 7]. Therefore, by analyzing the brain tissues of patients with PD with different Braak stages, we found that the hub genes have different expression levels in different stages; thus, examining the expression of the hub genes could help predict disease progression in PD. Through the above methods, we found that DEGs were significantly enriched in metabolic pathways and identified 10 hub genes that may be related to the pathogenesis of PD, namely, *GNG3*, *MAPK1*, *FPR1*, *ATP5B*, *GNG2*, *PRKACA*, *HRAS*, *HSPA8*, *PSAP*, and *GABBR2*. Among these, *HRAS* may have potential value in predicting the progression of PD.

## 2. Materials and Methods

### 2.1. Data Source

In this study, gene expression datasets were obtained from the GEO database (https://www.ncbi.nlm.nih.gov/geo/). A total of 109 human PD series cases were retrieved from the database. We chose human brain substantia nigra tissue specimens for analyses as they better reflect the true differences in gene expression in patients with PD. After careful review, we choose the series GSE42966 with clinical information on Braak staging. GSE42966 was based on the Agilent GPL4133 platform (Agilent-014850 Whole Human Genome Microarray 4x44K G4112F). Data were freely available online, and our study did not involve any experiments with humans or animals performed by any of the authors.

### 2.2. Data Processing of DEGs

The GEO2R online analysis tool (https://www.ncbi.nlm.nih.gov/geo/geo2r/) was used to detect DEGs between PD and normal samples, and the *P* value and |log FC| were calculated. Genes that met the cutoff criteria, *P* < 0.05 and |log FC| ≥ 1.0, were considered as DEGs. Genes with *P* < 0.05 and log FC ≥ 1.0 were considered as upregulated, and genes with *P* < 0.05 and log FC ≤ −1.0 were considered as downregulated genes.

### 2.3. GO and KEGG Pathway Analysis of DEGs

GO analysis [8] is a commonly used method for large-scale functional enrichment research, using which gene functions can be classified into biological process (BP), molecular function (MF), and cellular component (CC). KEGG is a widely used database that stores a large amount of data on genomes, biological pathways, diseases, chemical substances, and drugs. GO annotation and KEGG pathway enrichment analyses of DEGs in this study were performed using the Database for Annotation, Visualization, and Integrated Discovery (DAVID) tool (https://david.ncifcrf.gov/) [9]. In GO annotation analysis of DEGs, *P* < 0.05 and gene counts ≥20 were considered statistically significant; in the KEGG pathway enrichment analysis of DEGs, *P* < 0.05 and gene counts ≥10 were considered statistically significant.

### 2.4. PPI Network Construction and Hub Gene Identification

We used the Search Tool for the Retrieval of Interacting Genes (STRING) database (http://string-db.org/) to analyze PPI information [10, 11]. To evaluate the potential PPI relationship, the DEGs identified previously were mapped to the STRING database. PPI pairs were extracted with a combined score of >0.7. Subsequently, the PPI network was visualized using Cytoscape software (https://www.cytoscape.org/) [12]. Nodes with a higher degree of connectivity tend to be more essential for maintaining the stability of the entire network. CytoHubba, a plugin in Cytoscape, was used to calculate the degree of each protein node. We considered the top 10 identified genes as the hub genes in our study.

### 2.5. Identification of Hub Genes Affecting PD Progression

GraphPad Prism 8, graphing software that can perform data analysis and data visualization, was used to analyze the expression of these 10 hub genes at different stages of PD. We downloaded the clinical data of GSE42966 from the GEO database and extracted the expression data for the hub genes in Braak 3 and Braak 4 PD stages. Next, we divided the patients with PD into two groups according to different stages (Braak 3 and Braak 4) and finally used GraphPad Prism 8 to perform statistical analysis and plot graphs for the two groups of data.

## 3. Results

### 3.1. Identification of DEGs

**Figure 1:**
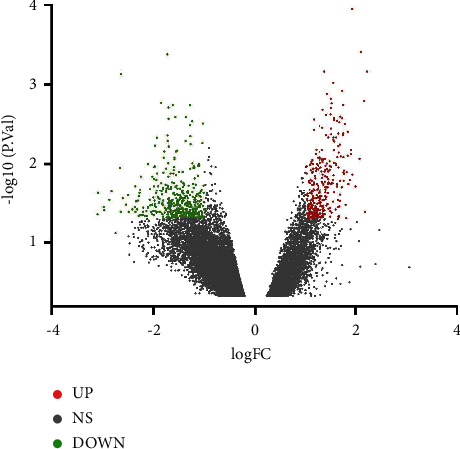
Volcano maps of all DEGs. Red represents upregulated genes, green represents downregulated genes, and gray represents genes that are not differentially expressed.

**Figure 2:**
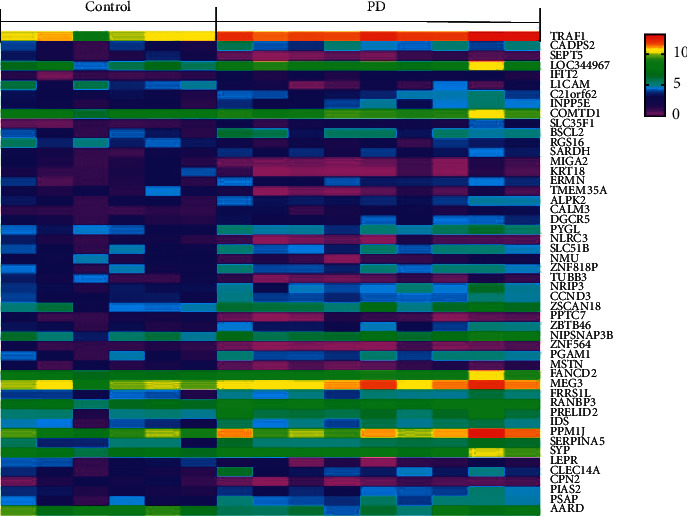
Heat map of the top 50 DEGs.

GSE42966 series was selected for the present study. GSE42966 contained nine PD samples and six normal samples. Based on the criteria of *P* < 0.05 and |log FC| ≥ 1.0, a total of 773 DEGs were identified from GSE42966, including 339 upregulated genes and 434 downregulated genes. All DEGs were identified by comparing the PD samples with the normal samples of the substantia nigra. A volcano map of all identified DEGs is shown in Figure 1. All DEGs were arranged in the ascending order of the *P* value since the smaller the *P* value, the more significant the difference. A heat map of the top 50 genes is shown in Figure 2. Information on the top 20 genes is presented in Table 1.

### 3.2. Functional Enrichment Analyses of DEGs

**Figure 3:**
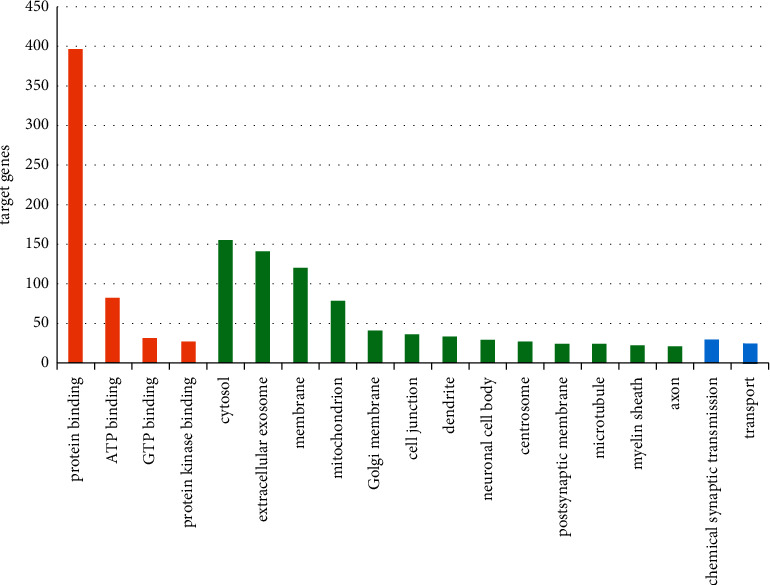
GO function enrichment analysis of DEGs. Orange represents MF, green represents CC, and blue represents BP. The numbers on the *y*-axis are gene counts.

**Figure 4:**
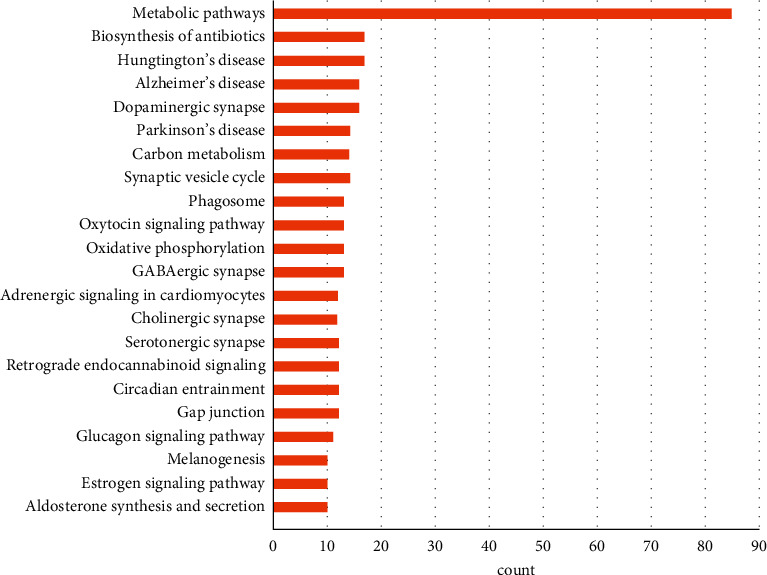
Enrichment analysis of the KEGG signaling pathway of DEGs. The numbers on the *x*-axis are gene counts.

GO function and KEGG pathway enrichment analyses for DEGs were performed using DAVID (Figures 3 and 4). The enriched GO terms were divided into CC, BP, and MF ontologies. The results of the GO analysis indicated that DEGs were mainly enriched in MF, including protein binding, ATP binding, GTP binding, and protein kinase binding. CC analysis showed that DEGs were significantly enriched in the cytosol, extracellular exosomes, membranes, mitochondria, Golgi membranes, cell junctions, dendrites, neuronal cell bodies, centrosomes, postsynaptic membranes, microtubules, myelin sheaths, and axons. BP analysis showed that DEGs were significantly enriched in synaptic transmission and transport. In addition, the results of the KEGG pathway analysis showed that DEGs were mainly enriched in pathways involved in metabolism, Huntington's disease, biosynthesis of antibiotics, dopaminergic synapses, Alzheimer's disease, synaptic vesicle cycle, carbon metabolism, PD, GABAergic synapse, oxidative phosphorylation, oxytocin signaling pathway, phagosome, gap junction, circadian entrainment, retrograde endocannabinoid signaling, serotonergic synapse, cholinergic synapse, adrenergic signaling in cardiomyocytes, glucagon signaling pathway, aldosterone synthesis and secretion, estrogen signaling pathway, and melanogenesis.

### 3.3. PPI Network Construction and Hub Gene Identification

**Figure 5:**
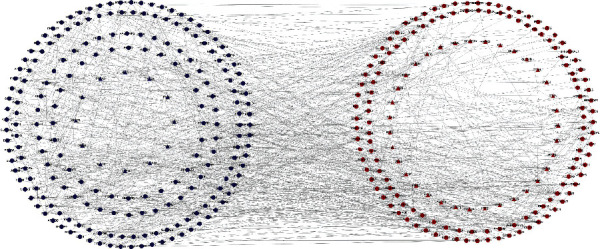
PPI network of DEGs. Blue represents downregulated genes, red represents upregulated genes, and the genes represented by triangles have more significant differences in gene expression than those represented by circles.

**Figure 6:**
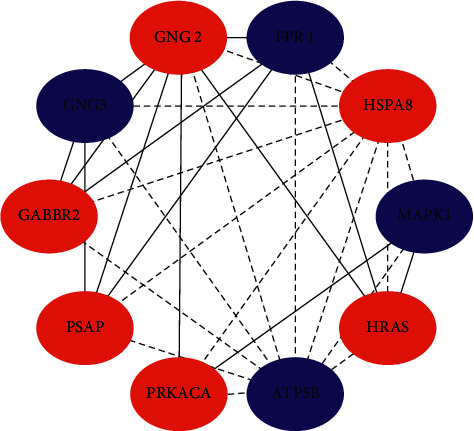
The top 10 hub genes in the PPI network. Blue represents downregulated genes, red represents upregulated genes, solid lines represent direct interactions between genes, and dotted lines represent indirect interactions between genes.

Protein interactions among the DEGs were predicted using the STRING tool. A total of 774 nodes were involved in the PPI network, as shown in Figure 5. The top 10 hub genes evaluated by the degree of connectivity in the PPI network were identified (Table 2 and Figure 6). The results showed that *GNG2* was the most outstanding gene with connectivity degree = 27, followed by *GNG3* (degree = 24), *PRKACA* (degree = 23), *HRAS* (degree = 23), *MAPK1* (degree = 22), *HSPA8* (degree = 20), *FPR1* (degree = 19), *ATP5B* (degree = 19), *PSAP* (degree = 18), and *GABBR2* (degree = 18). Among them, *GNG3*, *MAPK1*, *FPR1*, and *ATP5B* were downregulated in PD, and *GNG2*, *PRKACA*, *HRAS*, *HSPA8*, *PSAP*, and *GABBR2* were upregulated in PD. These 10 hub genes were significantly enriched in the six KEGG signaling pathways (Table 3), including the estrogen signaling pathway, serotonergic synapse, cholinergic synapse, chemokine signaling pathway, RAS signaling pathway, and pathways in cancer.

### 3.4. Identification of Hub Genes Affecting PD Progression

**Figure 7:**
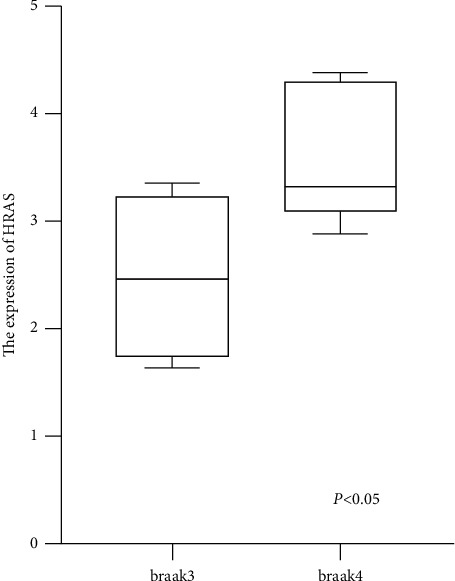
The expression of *HRAS* in brain tissues of patients with Braak 3 and Braak 4.

To investigate the values of the 10 hub genes in the progression of PD, we downloaded the clinical data of GSE42966 from the GEO database and extracted the expression data of the hub genes in Braak 3 and Braak 4 stages. We then divided the patients with PD into two groups according to different Braak stages (Braak 3 and Braak 4). Among them, Braak 3 group contained four samples, and Braak 4 group contained five samples. Finally, we performed statistical analysis for the data from the two groups and found that the expression of the upregulated gene *HRAS* in PD was higher in Braak 4 than in Braak 3, the difference being statistically significant (Figure 7). There were no significant differences in the expression of the other nine hub genes in different stages.

## 4. Discussion

PD is the second most common age-related neurodegenerative disease [13]. Genetic factors are known to play an important role in the pathogenesis of PD [3]. Till date, at least 28 genes are known to be related to the pathogenesis of PD [14], but the identity of genes involved in PD progression remains unclear, especially key genes that can predict disease progression. In this study, we identified a total of 733 DEGs that were significantly enriched in metabolic pathways. PPI network analysis found that 10 hub genes may be involved in the pathogenesis of PD. Among these, *HRAS* has the potential to predict the progression of PD.

The significant enrichment of DEGs in metabolic pathways as identified by this study suggests that this pathway may play an important role in the pathogenesis of PD. A previous study [15] demonstrated that multiple metabolic pathways are involved in PD. Tan et al. [16] also found the important value of metabolic pathways in the pathogenesis of PD by analyzing GEO dataset GSE72267, supporting the results from our study.

In this study, we also found that compared to the control group, the expression of *HRAS* was upregulated in patients with PD, with differences observed in expression between patients with PD with Braak 4 and Braak 3 stages, suggesting that *HRAS* expression may have the potential to predict the occurrence and progression of PD. *HRAS* is a member of the *RAS* oncogene family, and its mutations are common in a variety of cancers, including head and neck cancer, skin cancer, and hematopoietic cancers [17, 18]. However, the relationship between *HRAS* and PD has rarely been reported. Masuda et al. [19] found that the activation of the H-RAS/ERK signaling pathway plays an important role in the NO/ROS redox signal-mediated cell model of PD, suggesting that *HRAS* is involved in the pathogenesis of PD. *HRAS* is a GTPase protein that can activate the mTOR signaling pathway [20]. mTOR plays a key role in brain development, neuronal survival, synaptic plasticity, and memory formation [21] and is related to the pathogenesis of neurodegenerative diseases, especially PD [22]. mTOR is a serine/threonine kinase that is the core component of the mTORC1 and mTORC2 multiprotein complex. mTORC1 controls protein translation and autophagy [23]. Autophagy is the main degradation pathway that eliminates various aggregate proteins, and autophagy activation defects are common in many neurodegenerative diseases, such as PD [24]. Therefore, we speculate that *HRAS* overexpression may inhibit autophagy and promote the occurrence and development of PD by activating the mTOR pathway. In addition, results from our research showed that *HRAS* genes were significantly enriched in the following KEGG signaling pathways: estrogen signaling pathway, serotonergic synapse, cholinergic synapse, chemokine signaling pathway, RAS signaling pathway, and pathways in cancer. Among them, cholinergic synaptic signaling pathway dysfunction has been confirmed to be related to cognitive impairment in patients with PD [25], suggesting that *HRAS* may be involved in the pathogenesis of PD dementia; the specific mechanism remains to be confirmed by further studies.

Another study [26] showed that *HRAS* rs12628 is related to L-DOPA-induced dyskinesia (LID). LID is one of the most ineffective adverse reactions in the treatment of PD. Approximately 80% of patients with PD who receive levodopa treatment will develop LID within 5–10 years after starting DA replacement therapy [27]. This suggests that in-depth research on *HRAS* is also particularly important for the treatment of PD and may help prevent early and/or severe LID. *HRAS* could be potentially used as a target for the treatment of PD.

Prosaposin, encoded by the *PSAP* gene, is a multifunctional protein containing four saposin domains A–D, with neuroprotective, neurotrophic, and lysosomal function regulation effects [28]. Previous studies have shown that *PSAP* exerts a neuroprotective effect in PD models [29], and saposin deficiency can lead to age-dependent neurodegeneration [30]. Oji et al. [31] identified two *PSAP* saposin D-domain intron variants, rs4747203 and rs885828, in patients with autosomal dominant PD from Japan and Taiwan, which are related to susceptibility to PD. They also found that *PSAP* saposin D mutations can cause progressive motor decline and dopaminergic neurodegeneration in mice. Lin et al. [32] also found that the frequency of the rs142614739 variant of the *PSAP* saposin D domain in PD increased in the Eastern Chinese population, which is a risk factor for PD. Our research shows that the expression of the *PSAP* gene is upregulated in patients with PD and may be involved in the pathogenesis of PD, which is consistent with the above research results.

Our study showed that protein kinase A catalytic subunit (*PRKACA*) is highly expressed in the substantia nigra of patients with PD, and the KEGG enrichment pathway shows that it is involved in the dopamine and acetylcholinergic pathways. Chi et al. [33] analyzed DEGs in the GSE6613 dataset in the GEO database and found that *PRKACA* was upregulated in patients with PD and may be related to PD. Jia et al. [34] analyzed DEGs in different brain regions of the PD mouse model and found that *PRKACA* is one of the proteins in the PPI network that interacts closely with other proteins and may participate in the pathogenesis of PD by causing an imbalance in DA and acetylcholine. These conclusions are consistent with the results of this study.

*MAPK1* is a serine-threonine kinase belonging to the MAPK family. Excessive activation of MAPK can cause neuronal death in PD [35]. Previous studies have shown that *MAPK1* is associated with the progression of PD [36]. The accumulation of phosphorylated MAPK in the halo area of Lewy bodies also supports its pathogenic role in PD [37]. In addition, fibroblasts from patients with PD with the G2019S *LRRK2* mutation showed excessive activation of the MAPK1/3 pathway [38], and knockdown of long noncoding RNA SNHG1 can improve apoptosis, oxidative stress, and inflammation in PD models by inhibiting the miR-125b-5p/MAPK1 axis [39], which indicates that *MAPK1* is involved in the pathogenesis of PD. Our results show that, compared to the control group, *MAPK1* is abnormally expressed in PD and is the hub gene in PPI, suggesting its important role in the pathogenesis of PD, consistent with the aforementioned research results.

PD is well known to be caused by the progressive death of DA neurons in the substantia nigra of the midbrain, leading to a lack of DA in the striatum. Studies have shown that chronic DA depletion can enhance the transmission of GABA in the striatum, thus affecting cell and circuit activities, which are related to the pathogenesis of PD [40]. A study has shown [41] that patients with PD in Braak 3 exhibit an increased threshold of GABA medium spiny neurons, and patients with PD in Braak 4 exhibit decreased spontaneous GABA activity. The “GABA collapse” hypothesis proposed by Błaszczyk suggests that GABA imbalance plays an important role in the development and progression of PD and other neurodegenerative diseases. *GABBR2* is the main gene involved in GABA signal transduction and plays a vital role in maintaining the balance of excitation/inhibition in brain synapses [42]. Other studies have shown that *GNG3* may be related to the GABAergic synaptic pathway [43], and its destruction may lead to dysfunction of the GABAB1 receptor signaling pathway [44]. Our results suggest that *GABBR2* and *GNG3* are hub genes in the PPI network, and KEGG pathway enrichment analysis showed that they were significantly enriched in the GABAergic synaptic pathway, suggesting that they may participate in the pathogenesis of PD by affecting GABAergic synapse signaling.

Studies have shown that mitochondrial dysfunction is an important pathogenic mechanism that leads to motor dysfunction and damage to dopaminergic neurons in patients with PD [45, 46]. *ATP5B* is an important part of the mitochondrial respiratory chain complex and catalyzes the synthesis of ATP [47]. Our results show that the expression of *ATP5B* is downregulated in PD. It is speculated that PD may occur by affecting mitochondrial function, but the specific mechanism needs to be confirmed by further studies.

In addition, our research also showed that *GNG2* and *HSPA8* were upregulated in PD and were the hub genes in PPI. Lyu et al. [48] also found that *GNG2* may play a key role in the pathogenesis of PD by analyzing gene expression in different brain regions in a PD mouse model. Molochnikov et al. [49] found that *HSPA8* is abnormally expressed in blood of patients with PD compared to healthy individuals and has the potential to predict PD. These studies support our findings. Our study also shows that *FPR1* is downregulated in the substantia nigra of patients with PD and plays an important role as a hub gene. Recent studies have shown that *FPR1* activation can promote the proliferation and differentiation of nerve cells by activating reactive oxygen species and plays an important role in neurological diseases [50, 51]. Therefore, we speculated that it may participate in the occurrence of PD by inhibiting neuronal proliferation and differentiation. However, little is known about the role of these genes in PD, and further research is needed in the future.

This study has some shortcomings, including that the study only contains one series, and the number of samples is small. Further studies are warranted to confirm the results of this study. Therefore, we are going to establish in vitro and in vivo experimental models to verify our conclusions.

## 5. Conclusion

In conclusion, our data demonstrated that metabolic pathways play an important role in the pathogenesis of PD. Ten hub genes (*GNG3*, *MAPK1*, *FPR1*, *ATP5B*, *GNG2*, *PRKACA*, *HRAS*, *HSPA8*, *PSAP*, and *GABBR2*) may be involved in the pathogenesis of PD, among which *HRAS* may have potential value in predicting the progression of PD. However, due to the aforementioned limitations of the study, further investigation is needed in the future.

## Figures and Tables

**Table 1:** The top 20 of DEGs.

Gene	*P* value	Log FC	Description
*TRAF1*	0.000112	1.9436568	Up
*CADPS2*	0.000392	2.112701	Up
*SEPT5*	0.000417	−1.7267324	Down
*LOC344967*	0.000688	2.2360217	Up
*IFIT2*	0.00069	1.3968911	Up
*L1CAM*	0.000747	−2.6371341	Down
*C21orf62*	0.000961	1.572589	Up
*INPP5E*	0.001201	1.7463712	Up
*COMTD1*	0.001327	1.4499857	Up
*SLC35F1*	0.001518	1.5228036	Up
*BSCL2*	0.001626	2.1813648	Up
*RGS16*	0.001695	−1.8477099	Down
*SARDH*	0.00175	1.5392507	Up
*MIGA2*	0.001801	−1.2784036	Down
*KRT18*	0.001817	−1.6184519	Down
*ERMN*	0.001822	1.7714611	Up
*TMEM35A*	0.001953	−1.703526	Down
*ALPK2*	0.001989	1.5238576	Up
*CALM3*	0.002103	1.3650642	Up
*DGCR5*	0.002411	1.5177274	Up

*P* value was used for describing the significance of the expression difference between the PD group and the healthy control group. The smaller the *p* value was, the more significant the difference was.

**Table 2:** List of top 10 differentially expressed genes with higher degrees in the PPI network.

Gene	Description	Degree
*GNG2*	Up	27
*GNG3*	Down	24
*PRKACA*	Up	23
*HRAS*	Up	23
*MAPK1*	Down	22
*HSPA8*	Up	20
*FPR1*	Down	19
*ATP5B*	Down	19
*PSAP*	Up	18
*GABBR2*	Up	18

Degree was used for describing the importance of protein nodes in the network. The higher the degree was, the more important the nodes were in the network.

**Table 3:** List of KEGG pathways of hub genes.

Pathway ID	Pathway name	Count	*P* value	Genes
hsa04915	Estrogen signaling pathway	5	4.81*E* − 06	*PRKACA*, *HSPA8*, *GABBR2*, *MAPK1*, *HRAS*
hsa04726	Serotonergic synapse	5	7.60*E* − 06	*PRKACA*, *MAPK1*, *GNG2*, *GNG3*, *HRAS*
hsa04725	Cholinergic synapse	5	7.60*E* − 06	*PRKACA*, *MAPK1*, *GNG2*, *GNG3*, *HRAS*
hsa04062	Chemokine signaling pathway	5	5.86*E* − 05	*PRKACA*, *MAPK1*, *GNG2*, *GNG3*, *HRAS*
hsa04014	RAS signaling pathway	5	1.26*E* − 04	*PRKACA*, *MAPK1*, *GNG2*, *GNG3*, *HRAS*
hsa05200	Pathways in cancer	5	0.001050431	*PRKACA*, *MAPK1*, *GNG2*, *GNG3*, *HRAS*

KEGG: Kyoto Encyclopedia of Genes and Genomes.

## Data Availability

In this study, gene expression datasets were obtained from the GEO database (https://www.ncbi.nlm.nih.gov/geo/). After careful review, we chose the series GSE42966 with clinical information on Braak staging. GSE42966 was based on the Agilent GPL4133 platform (Agilent-014850 Whole Human Genome Microarray 4x44K G4112F). The data are freely available online, and our study did not involve any experiments with humans or animals performed by any of the authors.

## Abbreviations

PD: Parkinson's disease
DEGs: Differentially expressed genes
PPI: Protein-protein interaction
GNG3: G protein subunit gamma 3
MAPK1: Mitogen-activated protein kinase 1
FPR1: Formyl peptide receptor 1
ATP5B: ATP synthase F1 subunit beta
GNG2: G protein subunit gamma 2
PRKACA: Protein kinase A catalytic subunit
HRAS: HRas proto-oncogene, GTPase
HSPA8: Heat shock 70 kDa protein 8
PSAP: Prosaposin
GABBR2: Gamma-aminobutyric acid type B receptor subunit 2
LRRK2: Leucine-rich repeat kinase 2
VPS35: VPS35 retromer complex component
GEO: Gene Expression Omnibus
GO: Gene Ontology
KEGG: Kyoto Encyclopedia of Genes and Genomes
BP: Biological process
MF: Molecular function
CC: Cellular component
DAVID: Database for Annotation, Visualization, and Integrated Discovery
STRING: Search Tool for Recurring Instances of Neighbouring Genes
TRAF1: TNF receptor-associated factor 1
CADPS2: Calcium-dependent secretion activator 2
SEPT5: Septin 5
LOC344967: Acyl-CoA thioesterase 7 pseudogene
IFIT2: Interferon-induced protein with tetratricopeptide repeats 2
L1CAM: L1 cell adhesion molecule
C21orf62: Chromosome 21 open reading frame 62
INPP5E: Inositol polyphosphate-5-phosphatase E
COMTD1: Catechol-O-methyltransferase domain-containing 1
SLC35F1: Solute carrier family 35 member F1
BSCL2: Berardinelli–Seip congenital lipodystrophy type 2 protein
RGS16: Regulator of G-protein signaling 16
SARDH: Sarcosine dehydrogenase
MIGA2: Mitoguardin 2
KRT18: Keratin 18
ERMN: Ermin
TMEM35A: Transmembrane protein 35A
ALPK2: Alpha kinase 2
CALM3: Calmodulin 3
DGCR5: DiGeorge syndrome critical region gene 5
ATP: Adenosine triphosphate
GTP: Guanosine triphosphate
GMP: Guanosine monophosphate
GABA: *γ*-Aminobutyrate
mTORC: Mechanistic target of rapamycin complex
L-DOPA: Levodopa
LID: L-DOPA-induced dyskinesia
NO: Nitric oxide
ROS: Reactive oxygen species
DA: Dopamine.
